# Supplementary material for: Recurrent evolution of cryptic triploids in cultivated enset increases yield
Source: PLoS Genet. 2026 Jul 24;22(7):e1012241. doi: 10.1371/journal.pgen.1012241 (PMC13426944; doi:10.1371/journal.pgen.1012241)
Supplement: S1 Table — (DOCX) [file pgen.1012241.s001.docx]

**S1 Table Statistics of the *Ensete ventricosum* genome assembly**

|  | EnVent_Maze_1.0 (this study) | Bedadeti_annotated [19] |
| --- | --- | --- |
| *Assembly* |  |  |
| Assembly size (Mb) | 534.05 | 451.28 |
| Number of scaffolds | 806 | 47,742 |
| N50 / L50 (size in kb / count) | 57,407.2 / 5 | 21.1 / 6,010 |
| N90 / L90 (size in kb / count) | 35,910.5 / 9 | 4.0 / 23,334 |
| Largest scaffold (Mb) | 68.01 | 0.21 |
| % GC | 39.8 | 38.9 |
| % of repeat elements | 58.6 | - |
| BUSCO genome | C:98.6% [S:93.7%, D:4.9%], F:0.4% | C:84.2% [S:80.7%, D:3.5%], F:10.8% |
| *Gene annotation* |  |  |
| Number of protein-coding genes | 35,238 | 58,438 |
| BUSCO proteome | C:96.2% [S:91.5%, D:4.7%], F:1.7% | C:25.4% [S:24.3%, D:1.1%], F:22.4% |

BUSCO analyses have been carried out with the embryophyta_odb10 data set. C: complete genes, S: single copy, D: duplicated, F: fragmented genes.
