# Supplementary material for: Recurrent evolution of cryptic triploids in cultivated enset increases yield
Source: PLoS Genet. 2026 Jul 24;22(7):e1012241. doi: 10.1371/journal.pgen.1012241 (PMC13426944; doi:10.1371/journal.pgen.1012241)
Supplement: S2 Table — (DOCX) [file pgen.1012241.s002.docx]

**S2 Table Result summary of the Bayesian linear mixed-effects analysis**

|  | Estimate | Est. error | l-95% CI | u-95% CI | Rhat | Bulk_ESS | Tail_ESS |
| --- | --- | --- | --- | --- | --- | --- | --- |
| *Group-Level Effects* |  |  |  |  |  |  |  |
| sd(Intercept) | 1.36 | 0.32 | 0.74 | 2.02 | 1.00 | 5242 | 6046 |
| sd(logAge) | 0.81 | 0.21 | 0.40 | 1.24 | 1.00 | 4737 | 4782 |
| cor(Intercept,logAge) | -0.95 | 0.03 | -0.98 | -0.78 | 1.00 | 6720 | 6759 |
| *Population-Level Effects* |  |  |  |  |  |  |  |
| Intercept | 9.41 | 0.51 | 8.35 | 10.45 | 1.00 | 16578 | 18301 |
| logAge | 1.68 | 0.31 | 1.03 | 2.33 | 1.00 | 16699 | 18837 |
| pc1 | -0.10 | 0.06 | -0.22 | 0.03 | 1.00 | 16407 | 21673 |
| ploidy3n | -1.70 | 0.85 | -3.54 | -0.11 | 1.00 | 11609 | 15461 |
| pc2 | 0.51 | 0.10 | 0.30 | 0.71 | 1.00 | 16608 | 19874 |
| logAge:pc1 | 0.05 | 0.04 | -0.03 | 0.12 | 1.00 | 16794 | 21542 |
| logAge:ploidy3n | 1.21 | 0.52 | 0.22 | 2.32 | 1.00 | 12060 | 15965 |
| pc1:ploidy3n | 0.08 | 0.15 | -0.21 | 0.37 | 1.00 | 13008 | 17117 |
| logAge:pc2 | -0.33 | 0.07 | -0.46 | -0.19 | 1.00 | 16967 | 20557 |
| ploidy3n:pc2 | -0.47 | 0.24 | -0.95 | 0.00 | 1.00 | 11381 | 17030 |
| logAge:pc1:ploidy3n | 0.01 | 0.09 | -0.17 | 0.19 | 1.00 | 13677 | 18959 |
| logAge:ploidy3n:pc2 | 0.28 | 0.15 | -0.01 | 0.59 | 1.00 | 12000 | 17536 |
| *Family Specific Parameters* |  |  |  |  |  |  |  |
| sigma | 0.62 | 0.02 | 0.58 | 0.67 | 1.00 | 13669 | 18172 |

Estimate: median value of the posterior distribution. Est. error: median absolute deviation. l-95% CI: lower limit of the 95% confidence interval. u-95% CI: upper limit of the 95% confidence interval. Rhat: R-hat statistic for convergence diagnostic. Bulk_ESS: estimated bulk effective sample size. Tail_ESS: estimated tail effective sample size.
