# Supplementary material for: Recurrent evolution of cryptic triploids in cultivated enset increases yield
Source: PLoS Genet. 2026 Jul 24;22(7):e1012241. doi: 10.1371/journal.pgen.1012241 (PMC13426944; doi:10.1371/journal.pgen.1012241)
Supplement: S1 Fig — (DOCX) [file pgen.1012241.s003.docx]

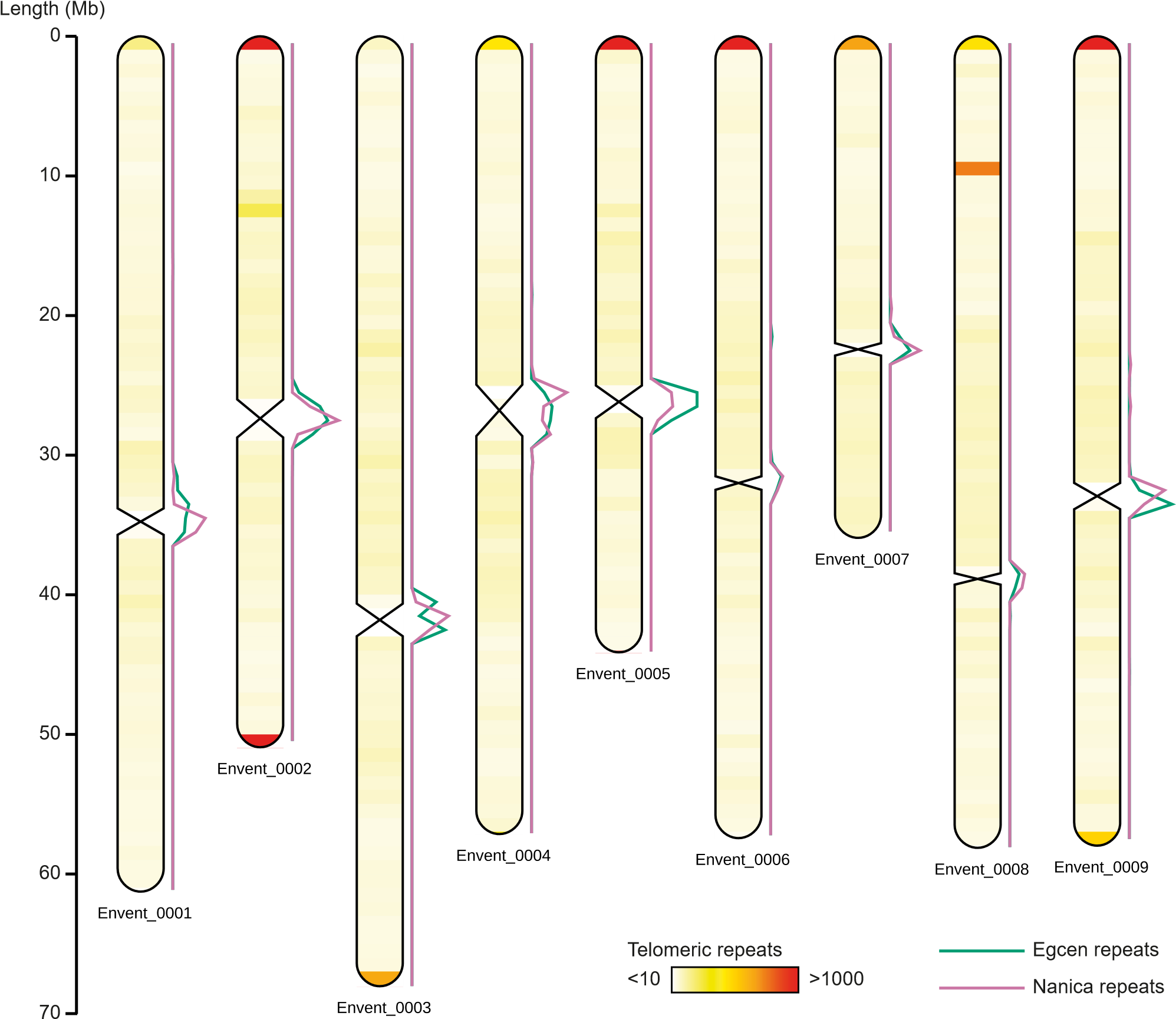


**S1 Fig Chromosome-scale assembly of the *Ensete ventricosum* genome.** The nine largest scaffolds are represented, with constriction points at the likely positions of centromeres. The number of canonical telomeric repeats is indicated by a color gradient inside each scaffold ideogram, and the relative abundance of Egcen and Nanica repeats, which are found in centromeric regions in other Musaceae, by colored lines on the right side of each ideogram. The number of all repeat types have been computed for 1 Mb windows along chromosomes. The maximum number of Egcen copies per window has been capped at 250, to allow for better readability.
