# Supplementary material for: Recurrent evolution of cryptic triploids in cultivated enset increases yield
Source: PLoS Genet. 2026 Jul 24;22(7):e1012241. doi: 10.1371/journal.pgen.1012241 (PMC13426944; doi:10.1371/journal.pgen.1012241)
Supplement: S2 Fig — (DOCX) [file pgen.1012241.s004.docx]

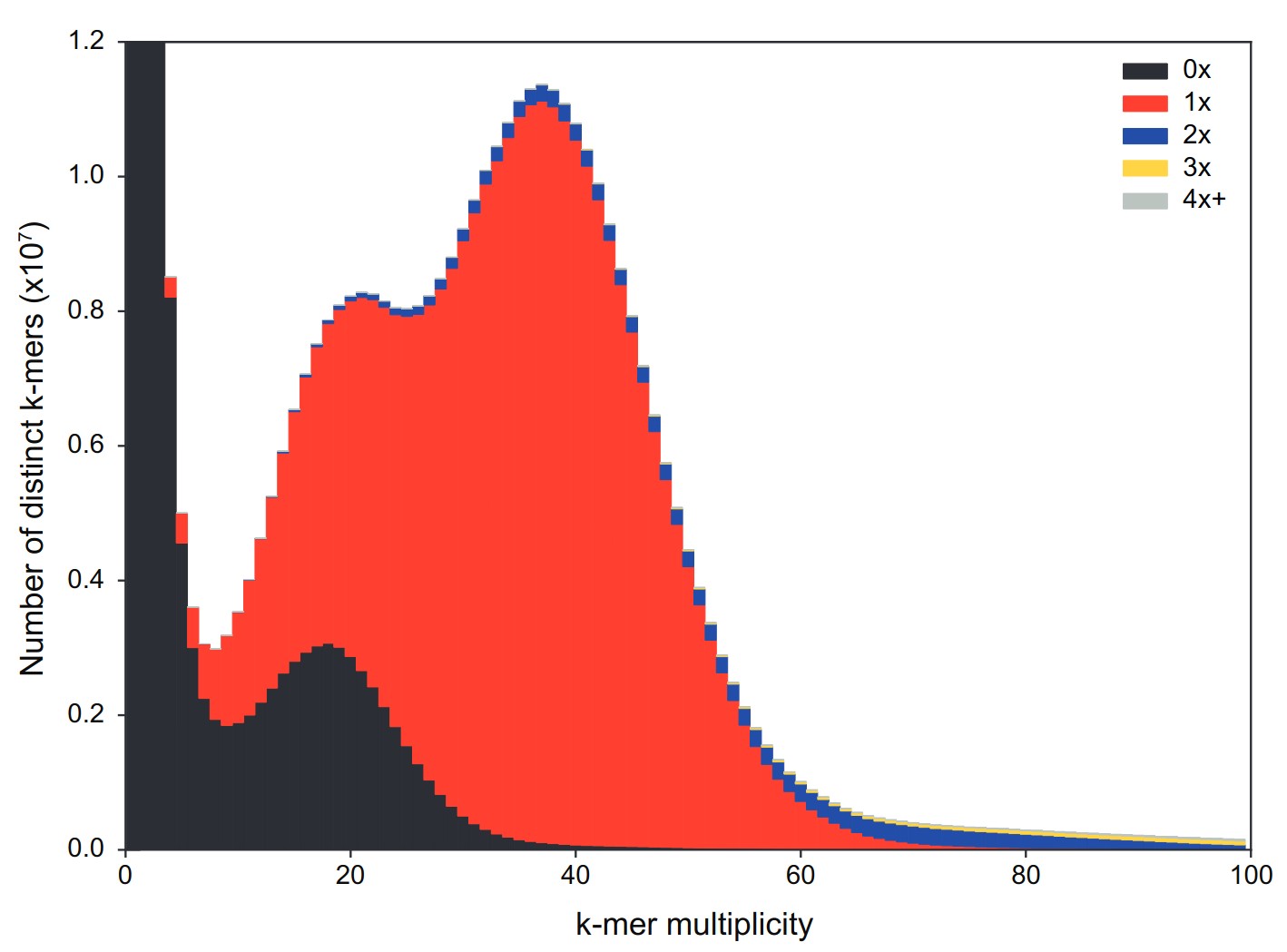


**S2 Fig K-mer content comparison between the CCS PacBio reads and the *Ensete ventricosum* genome assembly.** The k-mer spectrum (k = 21) for the PacBio reads is represented as a stacked histogram, with colored areas corresponding to k-mers absent from the assembly (0x), k-mers occurring once in the assembly (1x), k-mers occuring twice (2x), etc. Peaks at multiplicities 21 and 37 represent the heterozygous and homozygous content in the reads, respectively. The low proportion of homozygous k-mers found twice in the assembly (in blue) and the proportion of absent k-mer (in black) in the heterozygous peak are indicative of a good pseudo-haploid assembly.
