# Supplementary material for: Recurrent evolution of cryptic triploids in cultivated enset increases yield
Source: PLoS Genet. 2026 Jul 24;22(7):e1012241. doi: 10.1371/journal.pgen.1012241 (PMC13426944; doi:10.1371/journal.pgen.1012241)
Supplement: S3 Fig — (DOCX) [file pgen.1012241.s005.docx]

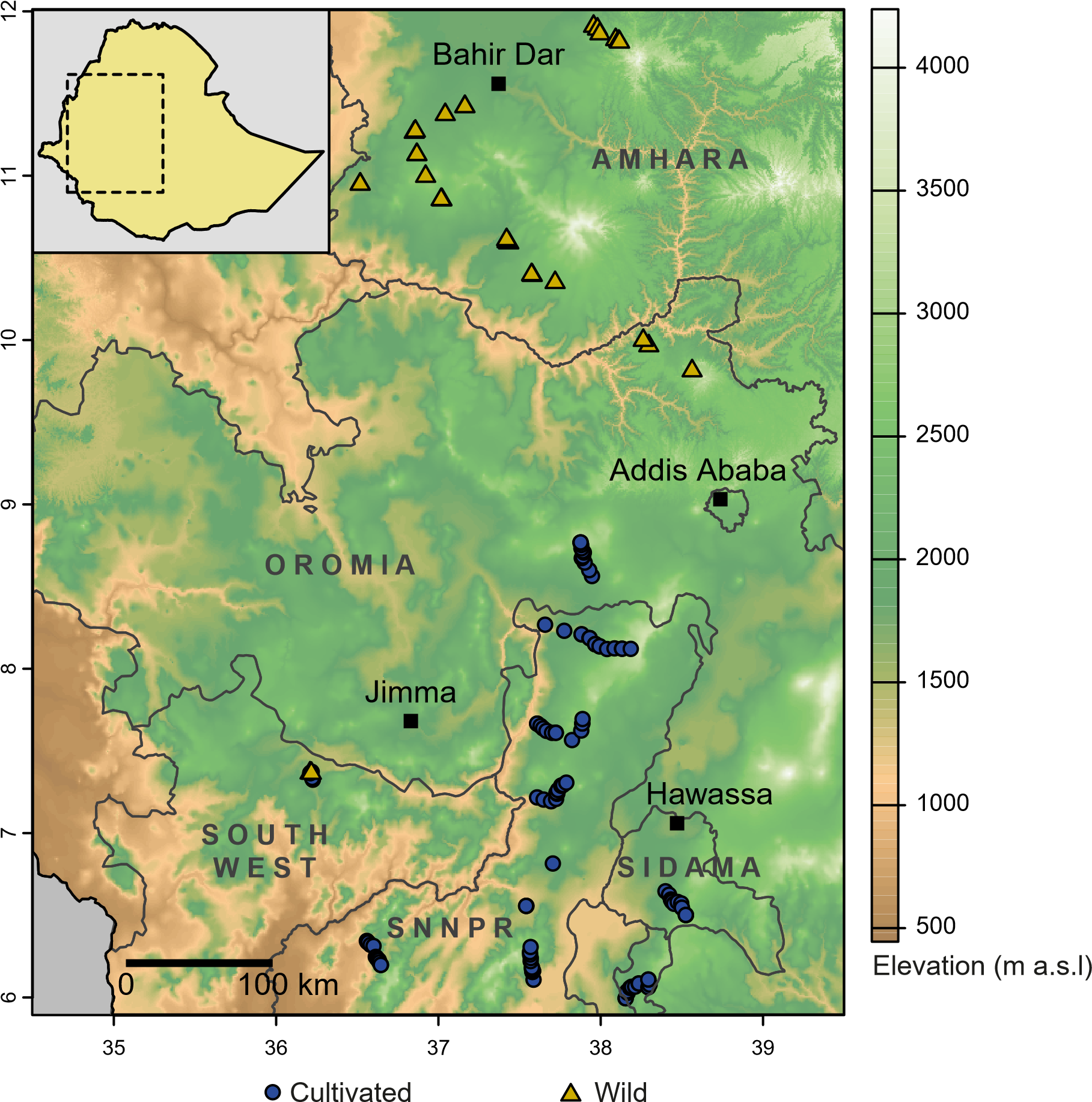


**S3 Fig** **Geographical distribution of the 658 cultivated and 65 wild individuals of *Ensete ventricosum* sampled in Ethiopia**. Cultivated individuals are represented by blue circles, wild ones by yellow triangles. Light gray lines delimit regional states, with their names indicated in bold. Major cities are represented by black squares. The inset at top left hand corner shows the total area of

Ethiopia, with the dashed square indicating the location of the enlarged map. The Ethiopia administrative boundaries shapefile was sourced from https://gadm.org.
