# Supplementary material for: Recurrent evolution of cryptic triploids in cultivated enset increases yield
Source: PLoS Genet. 2026 Jul 24;22(7):e1012241. doi: 10.1371/journal.pgen.1012241 (PMC13426944; doi:10.1371/journal.pgen.1012241)
Supplement: S4 Fig — (DOCX) [file pgen.1012241.s006.docx]

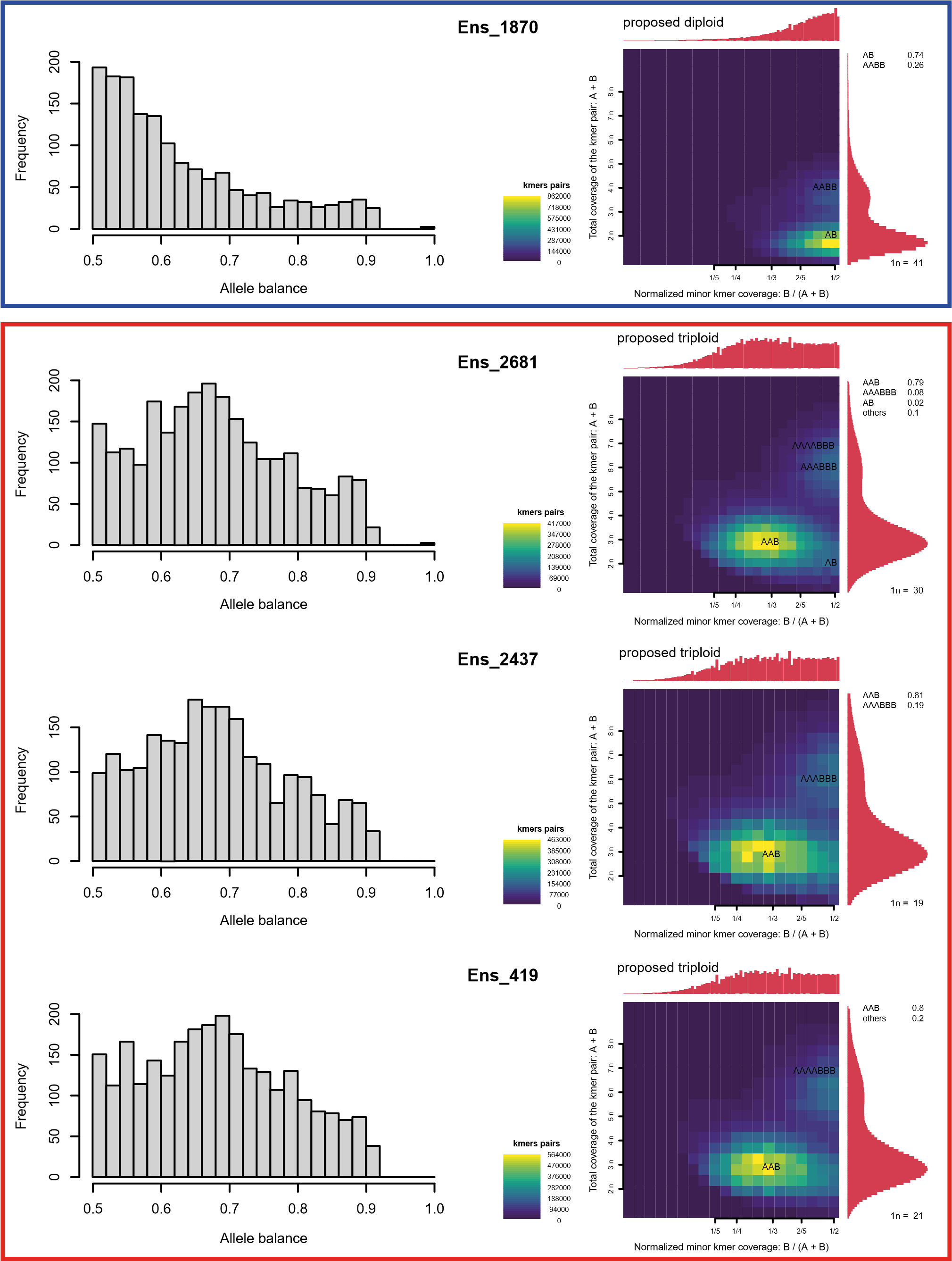


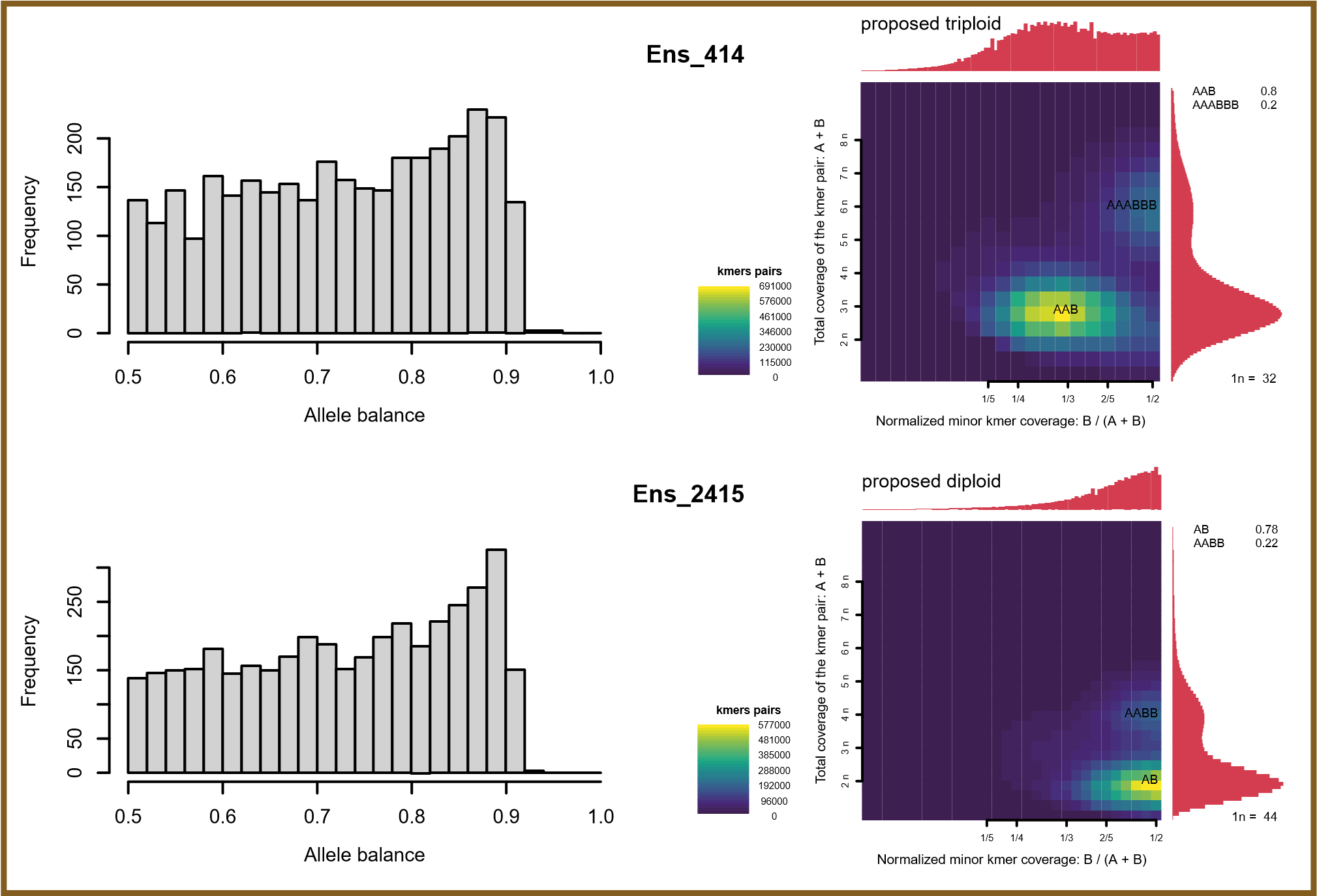


**S4 Fig Allele balance distributions and SmudgePlots for resequenced enset samples.** For each resequenced individual (rows), the allele balance distribution from the GRAS-Di data (left panels) is compared to the SmudgePlot k-mer analysis from the whole-genome resequencing data (right panels). In SmudgePlots, heatmap colors indicate the number of heterozygous (ie, differing by only one nucleotide) 21-mer pairs in each bin, from 0 in dark purple to the maximum value in bright yellow. Histograms represent the total coverage of k-mer pairs for each axis. The brighter "smudges" indicate the main ploidy of the sample, i.e. AAB for triploids or AB for diploids. Colored boxes around individuals indicate whether they were classified in the diploid (red), triploid (blue) or aberrant (brown) group on the basis of their allele balance distribution.
