# Supplementary material for: Recurrent evolution of cryptic triploids in cultivated enset increases yield
Source: PLoS Genet. 2026 Jul 24;22(7):e1012241. doi: 10.1371/journal.pgen.1012241 (PMC13426944; doi:10.1371/journal.pgen.1012241)
Supplement: S5 Fig — (DOCX) [file pgen.1012241.s007.docx]

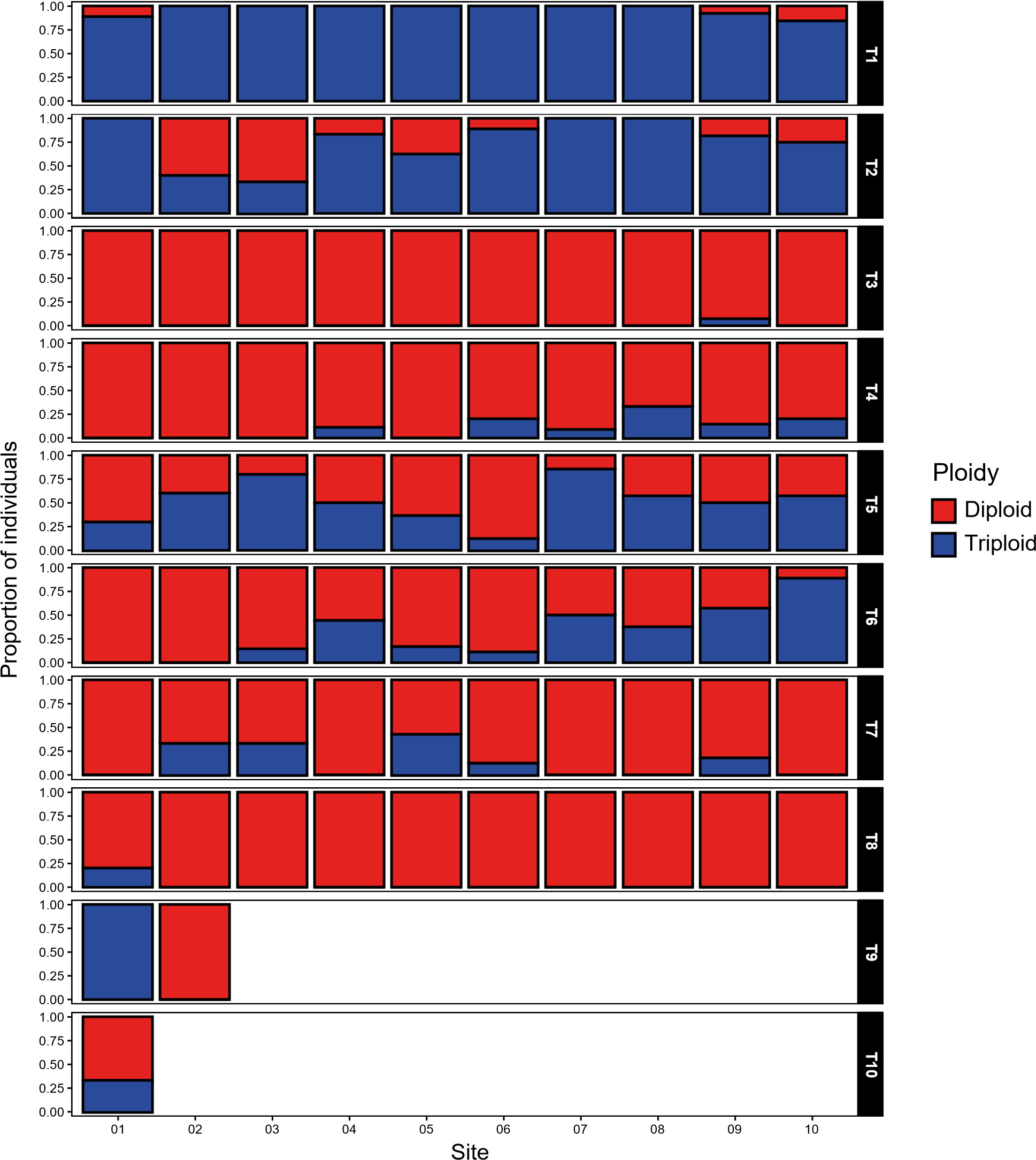


**S5 Fig Ploidy variation in cultivated enset in southwestern Ethiopia.** The proportion of individuals with a diploid or triploid cytotype is represented by a stacked barplot for each site (on the x axis) in each transect (in each panel). Sites within transects are ordered by altitude, with the lowest altitude to the left.
