# Supplementary material for: Recurrent evolution of cryptic triploids in cultivated enset increases yield
Source: PLoS Genet. 2026 Jul 24;22(7):e1012241. doi: 10.1371/journal.pgen.1012241 (PMC13426944; doi:10.1371/journal.pgen.1012241)
Supplement: S6 Fig — (DOCX) [file pgen.1012241.s008.docx]

A **Diploids** B **Triploids**

Frequency

0

2000

4000

6000

8000


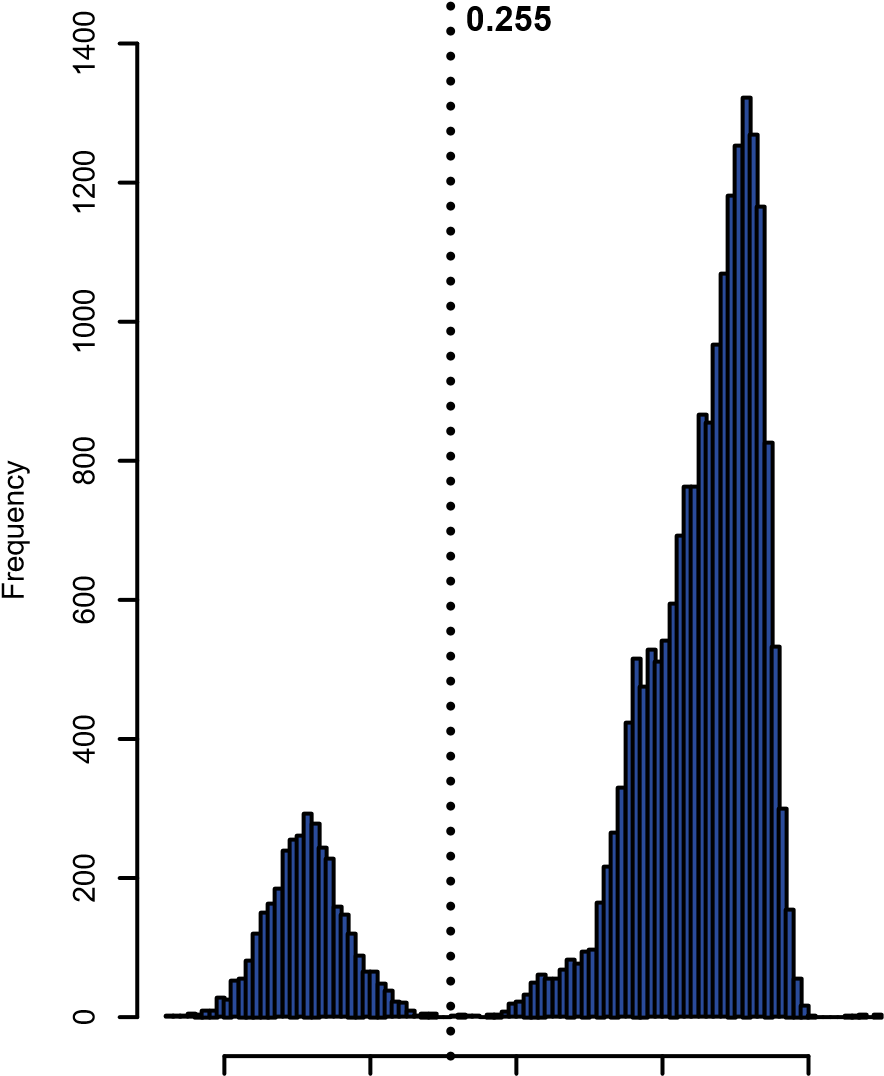


**0.003**

0.002 0.004 0.006 0.008 0.010 0.1 0.2 0.3 0.4 0.5

Pairwise euclidean distance Pairwise euclidean distance

**S6 Fig Distribution of pairwise Euclidean genetic distances in enset, between diploid (A) and triploid (B) individuals.** The dotted vertical line represents the distance threshold used to group samples into clonal lineages for each ploidy level, as the primary peaks correspond to pairs of samples with a very similar multi-locus genotype.
