# Supplementary material for: Recurrent evolution of cryptic triploids in cultivated enset increases yield
Source: PLoS Genet. 2026 Jul 24;22(7):e1012241. doi: 10.1371/journal.pgen.1012241 (PMC13426944; doi:10.1371/journal.pgen.1012241)
Supplement: S7 Fig — (DOCX) [file pgen.1012241.s009.docx]

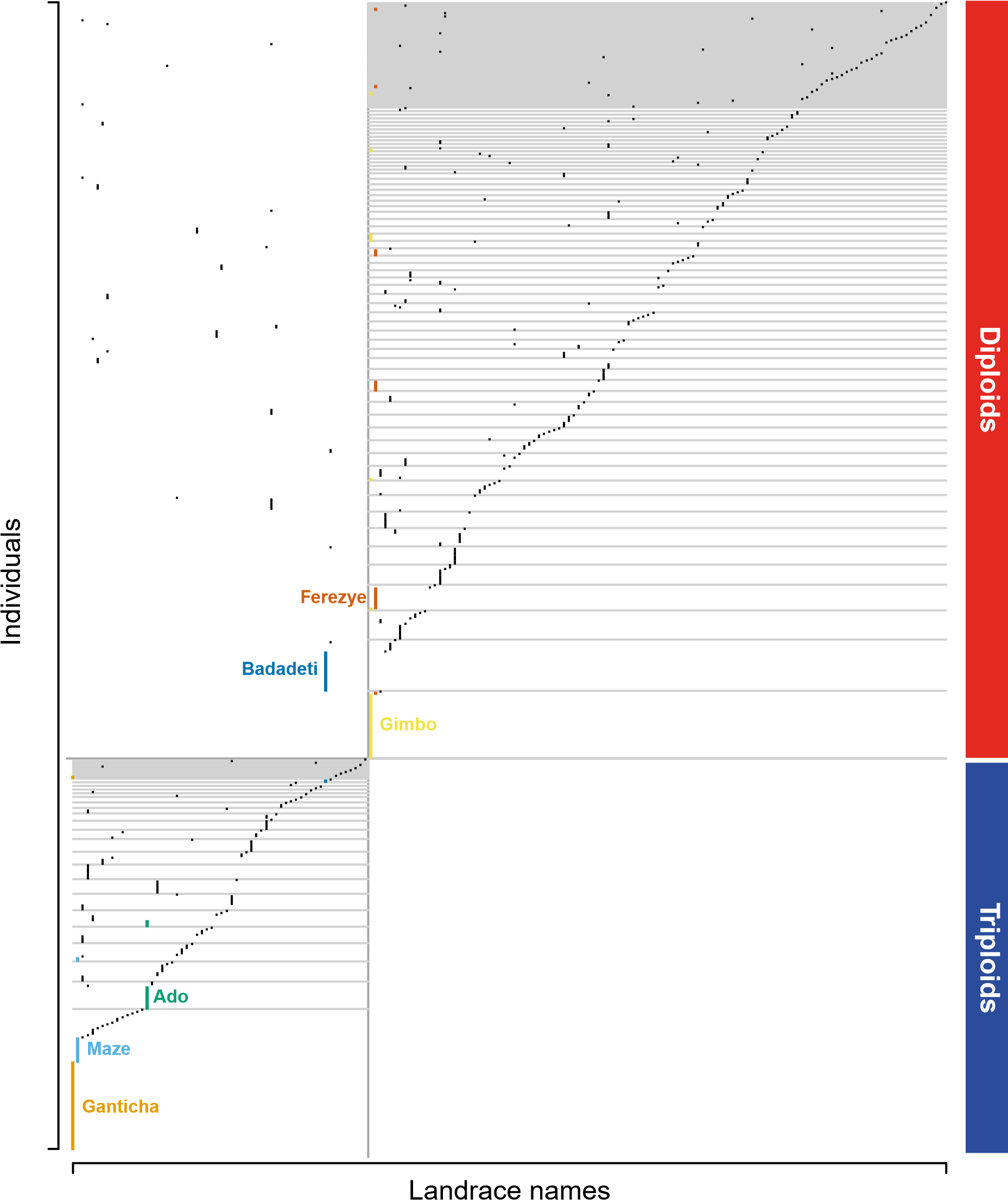


**S7 Fig Relationship between vernacular landrace names and clonal lineages for domesticated *Ensete ventricosum*.** Landrace names are on the x-axis and individuals on the y-axis. Clonal lineages are separated by vertical light gray lines on the left for triploids and on the right for diploids. For clarity, only the six most common varieties have been annotated with their name, and highlighted with a color matching their label.
