# Supplementary material for: Recurrent evolution of cryptic triploids in cultivated enset increases yield
Source: PLoS Genet. 2026 Jul 24;22(7):e1012241. doi: 10.1371/journal.pgen.1012241 (PMC13426944; doi:10.1371/journal.pgen.1012241)
Supplement: S8 Fig — (DOCX) [file pgen.1012241.s010.docx]

2

3

4

5

6

7

8

9

10

11

12

13

14

15

K

WAIC

(

x

10

⁶

)

A

1.35

1.45

1.55

1.65


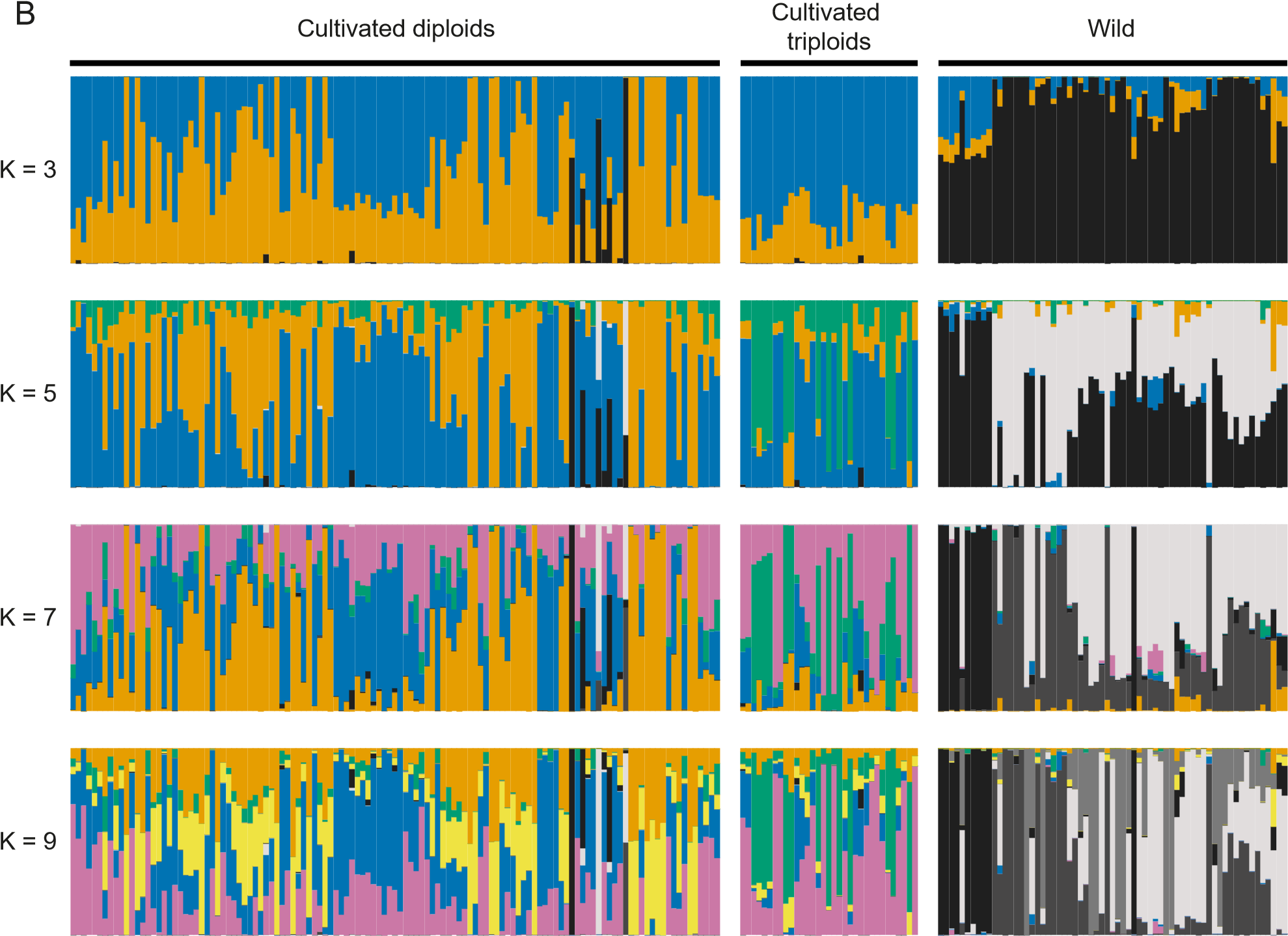


**S8 Fig Genetic structure of cultivated and wild enset, based on the entropy algorithm.** A: Values of the Watanabe-Akaike information criterion (WAIC) for each number K of populations. B: Individual membership coefficients in each genetic group for different values of K. Each individual is represented as a vertical bar, with colored segments corresponding to the estimated membership coefficients in each cluster.
