# Supplementary material for: Recurrent evolution of cryptic triploids in cultivated enset increases yield
Source: PLoS Genet. 2026 Jul 24;22(7):e1012241. doi: 10.1371/journal.pgen.1012241 (PMC13426944; doi:10.1371/journal.pgen.1012241)
Supplement: Attachment — Submitted filename: TriploidEnsetMS_PlosGenetics_responseReviews.docx [file pgen.1012241.s012.docx]

**>> Dear Reviewers and Editors,**

**Here is the revised version of our article entitled “Recurrent evolution of cryptic triploids in cultivated enset increases yield”. We thank you for the time you dedicated for our manuscript and for your comments and suggestions, which helped greatly improve it. In addition to minor corrections and the integration of missing information about the generation and treatment of the RNA-seq and Omni-C data in the Material and Methods, we significantly edited the Results and Discussion sections to follow the Reviewers’ suggestions.**

**You will find below our point-by-point response to Reviewers’ comments, written in blue. All line numbers are indicated for the revised version of the manuscript.**

**On behalf of all the authors,**

**Yann Dussert**

**Comments to the Authors:
Please note here if the review is uploaded as an attachment.**

**Reviewer #1:** Review of ‘recurrent evolution of cryptic triploids in cultivated enset increases yield’

In this paper, the authors state that, prior to their current study, enset was thought to be exclusively diploid. Enset (Ensete ventricosum, related to banana), the pseudostem and leaves of which are used as a main food source in Ethiopia, is an under-utilized and under researched orphan crop which nevertheless has great potential to enhance food security in sub-Saharan Africa. Here, the authors, using a newly assembled chromosome-scale reference genome, and sequence data from 723 wild and domesticated enset individuals from southwestern Ethiopia, show that, unexpectedly, around 20% of cultivated enset clones are triploid. The authors tested whether enset triploids offered yield advantages to farmers and concluded that indeed they do and that triploid cytotypes have been systematically (but unknowingly) selected by farmers because of trait differences (thicker pseudostems thus higher yield in triploids).

The study also showed that the triploids originated from multiple independent triploidization events, likely originating from sexual crosses between diploid domesticated individuals. Most of the triploid lineages were not related to each other and had different diploid parents.
All wild individuals that were studied and sequenced on the contrary, were diploid. Among cultivated individuals, 414 were diploid and 214 were triploid.

I truly enjoyed reading this paper. I think this paper is particularly interesting because:

- The results present a correlation between clonality and polyploidy, as has been observed in other angiosperms: this clonal agricultural propagation allowed avoiding the costs of infertility, resulting from polyploidy/triploidy. In this case, the advantages of being polyploid outweigh the disadvantages/cost of polyploids (more competition in the wild, then in an agricultural setting). This would also explain why all enset individuals sampled in the wild, were diploids.
- This study is a very nice and prime example of the ‘trajectory of polyploidization’ in relation to domestication in real time, and explaining/showcasing how, throughout time (going back 10.000 years) other crops as well probably have been domesticated (through the selection of polyploids because of trait differences, i.e., bigger fruits, bigger ears, bigger seeds, …).
- It tries to explain – a little speculative, but making sense, in my opinion – the latitudinal gradient for the presence of triploids.
- The paper also explains why selecting for triploids (because of higher yield, for instance) might not necessarily be a good thing on the long-term, because of decreased genetic diversity (due to a small number of triploid lineages being dominant).

I have gone over the paper twice but could not come up with any major concerns. In my opinion, this is an interesting study on a very interesting, and unexpected finding. Unexpected, but when considering what we know about polyploidy and the many polyploid crops that have been selected for since the domestication of food crops, also maybe not completely unexpected.

**>> We thank the reviewer for their positive comments and their appreciation of our article.**

**Reviewer #2**: This manuscript presents a compelling study on the recurrent evolution of triploidy in cultivated enset, demonstrating its association with increased yield and providing insights into polyploidy during domestication. The use of a new genome assembly and
large-scale resequencing is a strength, revealing unexpected triploidy in a species previously thought diploid. The integration of genomic, evolutionary, and phenotypic data is innovative, with implications for breeding and food security in Africa. The dataset is rich and the analyses are generally well executed, but several aspects of the study require clarification or additional support.

**>> We thank the reviewer for their positive comments.**

Line 119-128: The sequenced individual shows high genome-wide heterozygosity
(1.10%), yet a pseudo-haploid assembly was produced. Why was a phased haplotype-resolved assembly not attempted, especially given the potential for allelic differences in triploids? This could improve variant calling accuracy in heterozygous regions.

**>> We did try to obtain a diploid haploid-resolved genome, but the quality of the resulting assembly was much more fragmented than the pseudo-haploid one.**

Line 138: Ploidy determination is crucial for this study, as it underpins the identification of triploid lineages and their implications for yield and domestication. Currently, it relies on conventional genomic survey methods: allele balance (AB) and k-mer spectra analyzed via Smudgeplot, with confirmation in six resequenced individuals. If possible, providing more accurate flow cytometry results using fresh samples for representative individuals would strengthen the validation.

**>> We do agree that independently confirming our results using flow cytometry would have been a nice addition to our study, it was however not feasible. As mentioned in the Material and Methods section (l. 547-552), we did try this on our dried samples at the Kew facilities near London (UK), without success. The analysis of fresh plant material was not possible because 1) export regulations for living material from Ethiopia are quite restrictive and 2) no cytometer was available in Ethiopia.**

Lines 163-167: Triploids are more common in southern sites, correlated with latitude but not altitude. Is this gradient potentially influenced by unmeasured factors like disease prevalence or cultural preferences?

**>> There might indeed be unknown factors that could influence the presence of triploid ensets. We are not aware of any disease or pest gradients in the area. There are cultural differences between groups of farmers along latitude, but for now it is not clear how it would affect the proportion of triploids. One of our hypotheses (see l. 358 in the Discussion section) is that less intensive farming systems, with more semi-wild vegetation harbouring unmanaged enset populations, in the south could favor outcrossing, and consequently the production of triploid individuals. Other farmer practices could also impact the production of unreduced gametes (l. 356-358). However, it would not explain how triploids would persist and become more common, which is why we suggest in the Discussion that local adaptation could play a role in this gradient, or that we are observing an on-going process where some triploid clone (or at least the most dominant one) that have appeared in the south are in the process of being adopted further north.**

Lines 170–178; 501–507: Several key conclusions of the manuscript rely on the
inferred number of clonal lineages, the reduced diversity of triploids, and the
predominance of a single triploid clone. However, because the clone-delimitation threshold was chosen visually and applied separately to diploids and triploids, these inferences may be sensitive to threshold choice. The authors should assess whether the main conclusions are robust across a reasonable range of clone-delimitation thresholds.

**>> To choose a threshold for the determination of clonal lineages, we followed the method from (Arnaud-Haond et al., 2007), which is based on the fact that, in the distribution of genetic distances, the primary small peak with the lowest distances contains pairwise values between individuals from the same clones. In our case, the gap between this primary peak and the rest of the distribution was very clear. Note that the presence of multiple peaks in the distance distribution for diploid individuals is due to the presence of wild ensets in the analysis, as they display population genetic structure. Changing the chosen thresholds for clone delimitation using other values in the gap between peaks has only a very small effect, such as, for example for triploids, grouping with the most common clone an individual that represented a single clone.**

Lines 195-222: The low relatedness between diploid and triploid lineages suggests missing progenitors. Is this potentially due to incomplete sampling across Ethiopia, or could ancient triploidization events explain it? Additional phylogenetic modeling (e.g., using coalescent simulations) might clarify the timing of these events.

**>> It is still unclear whether the absence of the diploid parents of triploids is due to incomplete sampling or an ancient triploidization event. Though our sampling is quite comprehensive and is covering most of the main enset cultivation area, we cannot yet reject the possibility that progenitors do still exist and have not been collected, either because they are rare, or found in the margins of the cultivation area.**

**Our interpretation of the origins of triploids is particularly informed by the high degree of relatedness between triploids and the majority of domesticated diploids (Figure 2A). Hence although there are indications of some gene flow from wild diploids into the domesticated diploid gene pool, the triploids appear at first sight to have been generated from within the main domesticated group. That interpretation, and the need for triploids to be artificially propagated to persist, puts a limit on the date of triploidization perhaps about 8,000 years or 800 generations. Interpreting rates of coalescence on that time scale would be challenging without being able to obtain full phased haplotypes, although this might be possible with long read data in the future.**

Lines 230-245: The Bayesian mixed-effects model shows triploids have 42–75% larger pseudostem volumes, but measurements were conducted in situ. It remains unclear how confounders such as soil fertility or farmer-specific practices were fully
accounted for beyond bioclimatic PCA. Given the impracticality of common garden
experiments for enset due to its long growth cycle, the authors might consider
assembling panels of geographically proximate diploid and triploid individuals
sharing similar environmental conditions to repeat the yield comparison, which
would help isolate ploidy effects from environmental heterogeneity. And the evidence supporting a yield increase associated with triploidy is not yet conclusive. I therefore suggest that the authors moderate this claim and remove “increase yield” from the title.

**>> As some of those confounding factors could not be measured, it is true that they could not be accounted for in our model, as mentioned l. 298-302 of the Results. We do agree that, in the short term, it might be beneficial to focus on sampling diploid and triploid individuals from the same area (even the same farm when possible) to try to limit the impact of confounders.**

**In terms of analysis, we did implement an approach similar to the reviewer’s suggestion: in addition to our inference using a Bayesian linear mixed-effects model, we used a matching approach (a form of subset selection)** [**(D. E. Ho et al., 2007)**](https://www.zotero.org/google-docs/?Czu41B) **to generate control (2n) and treatment (3n) groups with balanced covariates, as implemented in the R package MatchIt** [**(D. Ho et al., 2011)**](https://www.zotero.org/google-docs/?fYDj3X)**. Specifically we employed a combination of full and exact matching, with ploidy as the treatment and transect, site and plant age as covariates. This generated subclasses with weighted samples from the same location (seeking to minimise unmeasured agroecological and management variables), with the same age, but with differing ploidy. We then fitted a linear regression model with pseudostem volume as the response variable with ploidy, transect, site and age as additive predictors and included weights across all samples. Similarly to the mixed-effects model, this analysis found a significant (p-value < 0.001) higher pseudostem volume for triploids on average. We decided to not include this analysis in the manuscript because 1) it seemed redundant and 2) the mixed-effects model allowed us to take into account other important variables such as relatedness between clones or the quantitative variation of environmental variables through the use of principal components.**

**For the comment on yield, we note that the entire pseudostem tissue is processed into the principal enset-derived food product, kocho. Therefore we believe that pseudostem volume is a very good predictor of resulting yield. This is corroborated by** [**(Mellisse et al., 2017)**](https://www.zotero.org/google-docs/?EOOTul)**, who showed that pseudostem size measurements are correlated with kocho yield with an R2 of ~0.85. Given that other studies have determined that the yield of some minor enset products (fiber and bulla) are harder to predict from plant trait measures, we clarify in the text that we are specifically referring to kocho yield. We now make this point clearly in the Results section for the phenotype analysis (l. 264-266) to explain the relationship between pseudostem volume and yield.**

Minor points:

- Line 25: I think “polyploids” is sufficiently familiar to the audience and does not require explanation in parentheses.

**>> We chose to keep the short explanation of “polyploids” in the abstract, to ensure comprehension from non-experts.**
- Line 81: the citations of the seven referred studies is missing.

**>> Sentence rewritten for clarity, as this is one study summarizing the chromosome counts for seven species, not seven separate references.**
- Line 214: estimation of kinship among individuals of different levels of ploidy is not common task, the author should briefly explain methods used and add citations. And citations should also be added in figure legend of 2B.

**>> We added a brief sentence to explain our approach in the Results (l. 242-244), and corresponding references in the legend of Fig. 2.**

**Reviewer #3**: The manuscript describes the development of an improved long read assembly for an orphan crop, enset, a clonally propagated starch crop, which sustains a significant population in Ethiopia. Notably the authors have determined that what was believed to be a purely diploid species has domesticated triploid lineages among its ranks. This is a novel and interesting finding; the non-random distribution of the triploid lines across the different latitudes is clear and provides a nice discussion point.

**>> We thank the reviewer for their positive comments.**

The authors are left to speculate as to when these triploid clones appeared, would it be possible to carry out Ks analyses of their limited whole genome resequencing data to attempt to gain some insights into this?
**>> We think that the Ks approach, based on the synonymous substitution rate, is not compatible with our data. First, triploidization events in enset probably happened recently and post-domestication, which means that a Ks analysis would not have much power. Second, Ks is supposed to be calculated using fixed substitutions, while our SNP dataset includes intra-species polymorphisms which are not fixed.**

I have listed a few comments and questions related to the results and materials and methods below.
Results
The results indicates that the genome was sequenced from the seed of a cultivated variety Maze, yet in the M&M it is stated that the line sequenced was derived from a cross between Maze and an unknown line, does that mean the genome is for an F1 line? The assembly based on the limited information provided in the supplementary data seems excellent considering the relatively low PacBio coverage and the apparent heterozygosity in the sequenced line.

**>> The sequenced individual was derived from the seed of a plant of the Maze variety, which is consequently the female parent, found in a cultivated field. We do not know the pollen donor (male parent), and we added this information in the Results for clarity (l. 148). It is likely that the pollen donor plant is from a different variety, but we do not have any real evidence that the sequenced individual is an F1 hybrid.**

The data analyses appears robust but I did wonder about the genomic distribution of SNP loci after filtering? Did they see any biased selection for particular regions of the genome in the different lineages?

**>> We did not observe any genomic regions with a lack or very low density of SNPs, except for very repeated regions (e.g. centromeres). Additionally, we did not detect any region with an excess of missing data for a particular clone.**

The statement ‘Thus farmers unconsciously classify plants according to their ploidy based on phenotype alone’ is a little misleading. It appears the farmers classify all their plants based on phenotype, so they will separate all plants, irrespective of ploidy, based on their phenotype.

**>> We agree with the reviewer, and consequently this sentence has been discarded.**

Line 190: Maze in T2 is identified as a triploid clone. I assume this is different from the Maze line that was sequenced, which was a diploid?

**>> This is the same Maze variety, which was the one of the female parent of the sequenced individual used for genome assembly, as mentioned above. Unfortunately, we did not keep any tissue from this parent, so we could not test whether or not it was also triploid. It is however likely, since all Maze individuals were triploid in our study. Triploid individuals might still be able to produce reduced (1n) gametes, which could explain why the sequenced offspring is diploid. We now briefly mention this in our Discussion about potential loss of fertility for triploids (l. 386-387).**

The authors discuss the possible link between the increased ploidy and increased yield; although the phenotype data is not perfect for all the reasons they listed, I did wonder if the triploid clone which appeared to be predominantly cultivated (36% of the triploids) confirmed this theory – was the pseudostem much larger in these lines?

**>> Yes, the pseudostem volume for the most represented triploid clone was larger than for diploids, and also higher than the average values for triploids. We illustrate this with the figure below, which shows raw values for the pseudostem volume in function of the age of the plant for all diploids (in red), all triploids (blue), and the predominant clone (black). Trend lines are results of fitted simple linear models.**

Materials and Methods

The methodology for the generation of the Omni-C library is generic and does not provide sufficient detail, perhaps provide a reference or indicate that this was completed by a service provider.
What was the length of the Illumina reads for the Omni-C data, presumably they were paired-end?
What was the age of the plants used to generate the RNASeq data? They were grown in a greenhouse?
How much RNASeq data was generated?
HiRise – version?
How were the Omni-C reads pre-processed?
Omni-C reads aligned with bwa – with default parameters, version?

**>> Indeed, the sequencing and assembly for the genome reference have been carried out by a service provider, Dovetail Genomics. It is now mentioned in the Materials and Methods section (l. 428 and 442). We also added information about the Omni-C and RNAseq data in the manuscript (l 415-417 and paragraph starting l. 418). Unfortunately, some information was not made available to us, namely the version of HiRise and bwa used for the scaffolding step.**

**Reviewer #4**: This manuscript presents a comprehensive population genomic analysis of Ensete ventricosum, supported by a newly-assembled chromosome-scale reference genome and extensive sampling (723 wild and domesticated enset individuals). The authors report the unexpected presence of widespread cryptic triploid lineages in cultivated enset and demonstrate that triploidy is associated with increased yield. The study is novel and highly relevant for understanding crop domestication and food security in Sub-Saharan Africa.

**>> We thank the reviewer for their positive comments.**

However, several aspects require clarification and improvement, particularly regarding the interpretation of triploid origins, ecological explanations, and the logical organization of the results and discussion sections. To further enhance your manuscript, we offer the following suggestions:
Major:
1.Mechanisms of triploid origin: The authors conclude that triploids arose from “multiple independent events among cultivated diploids” (lines 220-221). While the population genetic analyses support multiple origins, the underlying mechanism remains unclear. It is essential to further discuss whether these triploids originated via unreduced (2n) gametes within diploid populations or through hybridization between individuals of different ploidy levels. At present, the interpretation remains largely inferential; a more cautious discussion or additional allele dosage analysis would significantly strengthen this conclusion.

**>> This is indeed a good point, as the high relatedness between some pairs of triploids could theoretically be explained by parent-child relationships. We added this possibility in the Results (l. 247-251), although we note that involvement of triploid clones in the production of other triploid lineages is less likely since triploid plants are generally not or less fertile.**

2.Limited explanation for the latitudinal gradient in triploid frequency: The study identifies a strong correlation between triploid frequency and latitude (lines 163-166), but the explanations provided (e.g., temperature effects on unreduced gamete formation, differences in farming practices) remain speculative and are not directly supported by data. The authors are encouraged to either provide additional analyses linking specific environmental variables to triploid frequency or clearly frame these explanations as hypotheses rather than definitive conclusions.

**>> Following this comment, this part of the Discussion (l. 352-367) has been rewritten to clearly specify that our possible explanations for the observed gradient in triploid frequency are merely hypotheses. We do not think we currently have the data to link the proportion of triploids to the different possible described factors that could influence this gradient.**

3.Genotype-environment interaction: The manuscript shows that triploids may have higher pseudostem volume (line 230), but it remains unclear whether this advantage is consistent across environments. It would be valuable to discuss whether triploids exhibit environment-specific advantages, their phenotypic stability across different altitudes, and the potential for genotype × environment interactions.

**>> We added a short sentence (l. 278-282) clarifying that, while the growth rate of diploid ensets varied along the environment when looking at PC2 (correlated with latitude), it was not the case with triploids, which seemed to have consistent growth rate along PC2. We decided to not overstate the importance of this result, since latitude was also correlated with the proportion of triploids found on farms - notably the number of triploids in one of the terciles of PC2 was quite low.**

4.Genetic diversity and agricultural risk: The authors note that the dominance of a few triploid clones may reduce genetic diversity (lines 319-324). This is an important point but could be expanded. The discussion would benefit from a more explicit comparison with other clonally propagated crops.

**>> Starting l. 369, we now mention how the incorporation of individuals originating from sexual crosses in other clonal crops are important to generate new genetic diversity, and consequently how the predominance of triploid clones in enset could narrow its genetic base and adaptive potential.**

5.Structure and logical flow of results and discussion: The results section would benefit from improved logical organization, with clearer connections between figures and findings. Similarly, the discussion is somewhat fragmented, with limited progression between paragraphs. The current structure relies heavily on simple transitions (e.g., “First”, “Finally”) rather than developing a cohesive narrative. The authors are strongly encouraged to strengthen the logical flow, enhance transitions between core ideas, and more effectively align the discussion with the study's primary findings to highlight their broader implications.

**>> The Results and Discussion sections have been extensively edited to better connect our results and the figures illustrating them, as well as to improve the structure of the Discussion.**

Minor points:
1.Language and grammar:
a)Line 26: “robustness to stress and to disease” → “robustness to stress and disease”
b)Line 38: “sub-saharan Africa” → “Sub-Saharan Africa”
c)Line 41: “a species entire genome” → “a species’ entire genome”
d)Line 65: “masking of deleterious mutations” → “masking by deleterious mutations”
e)Line 72: “polyploidy crops” → “polyploid crops”
f)Line 98: simplify to “a high-yielding, ecologically resilient and flexible crop”
g)Line 145: “suggests” → “suggested”; “not selfing” → “rather than selfing”
h)Line 257: “are possible confounding effects” → “were possible confounding effects”
i)Line 259: “differences of” → “differences in”
2.Formatting and notation:
a)Line 101: “1,500 to 3,000 m” → “1,500-3,000 m”
b)Ensure consistent formatting of k-mer throughout the manuscript
c)Italicize all p-values (e.g., lines 164, 166, 167, 187)
d)Define abbreviations such as FST and CI at first mention
e)Line 357: “DNAse I” → “DNase I”
f)Ensure consistent use of quotation marks (e.g., lines 99 and 555)
3.Terminology consistency:
a)Use “clonal propagation” consistently instead of alternating with “vegetative propagation”
b)Ensure consistent usage of key technical terms throughout the manuscript
c)Standardize formatting conventions (e.g., gene names in italics)
4.Figures and presentation:
a)Provide clearer explanation of interaction effects in Figure 3
b)Strengthen connections between figures and corresponding text in the results section
5.References:
Please ensure that all references strictly follow the journal’s formatting guidelines.

**>> We modified the manuscript to follow all the suggestions from these minor points, except for point 1.d. which we think would change the meaning of the sentence and would not be correct.**

**REFERENCES**

[**Ho, D. E., Imai, K., King, G., & Stuart, E. A. (2007). Matching as nonparametric preprocessing for reducing model dependence in parametric causal inference. *Political Analysis*, *15*(3), 199–236. https://doi.org/10.1093/pan/mpl013**](https://www.zotero.org/google-docs/?Y2RHJm)

[**Ho, D., Imai, K., King, G., & Stuart, E. A. (2011). MatchIt: Nonparametric preprocessing for parametric causal inference. *Journal of Statistical Software*, *42*(8), 1–28. https://doi.org/10.18637/jss.v042.i08**](https://www.zotero.org/google-docs/?Y2RHJm)

[**Mellisse, B. T., Descheemaeker, K., Mourik, M. J., & Ven, G. W. J. van de. (2017). Allometric equations for yield predictions of enset (*Ensete ventricosum*) and khat (*Catha edulis*) grown in home gardens of southern Ethiopia. *Annals of Applied Biology*, *171*(1), 95–102. https://doi.org/10.1111/aab.12350**](https://www.zotero.org/google-docs/?Y2RHJm)
