# Supplementary material for: Recurrent evolution of cryptic triploids in cultivated enset increases yield
Source: PLoS Genet. 2026 Jul 24;22(7):e1012241. doi: 10.1371/journal.pgen.1012241 (PMC13426944; doi:10.1371/journal.pgen.1012241)
Supplement: Peer review history

# Peer review: Decision Letter 0

**DOI:** 10.1371/journal.pgen.1012241.r001

**Authors:** Justin Fay, Jae Young Choi

31 Mar 2026

PGENETICS-D-26-00202

Recurrent evolution of cryptic triploids in cultivated enset increases yield

PLOS Genetics

Dear Dr. Dussert,

Thank you for submitting your manuscript to PLOS Genetics. After careful consideration, we feel that it has merit but does not fully meet PLOS Genetics's publication criteria as it currently stands. Therefore, we invite you to submit a revised version of the manuscript that addresses the points raised during the review process.

Please submit your revised manuscript by May 30 2026 11:59PM. If you will need more time than this to complete your revisions, please reply to this message or contact the journal office at plosgenetics@plos.org. When you're ready to submit your revision, log on to https://www.editorialmanager.com/pgenetics/ and select the 'Submissions Needing Revision' folder to locate your manuscript file.

Please include the following items when submitting your revised manuscript:

* A letter that responds to each point raised by the editor and reviewer(s). You should upload this letter as a separate file labeled 'Response to Reviewers'. This file does not need to include responses to any formatting updates and technical items listed in the 'Journal Requirements' section below.

* A marked-up copy of your manuscript that highlights changes made to the original version. You should upload this as a separate file labeled 'Revised Manuscript with Track Changes'.

* An unmarked version of your revised paper without tracked changes. You should upload this as a separate file labeled 'Manuscript'.

If you would like to make changes to your financial disclosure, competing interests statement, or data availability statement, please make these updates within the submission form at the time of resubmission. Guidelines for resubmitting your figure files are available below the reviewer comments at the end of this letter.

We look forward to receiving your revised manuscript.

Kind regards,

Jae Young Choi

Academic Editor

PLOS Genetics

Justin Fay

Section Editor

PLOS Genetics

Aimée Dudley

Editor-in-Chief

PLOS Genetics

Anne Goriely

Editor-in-Chief

PLOS Genetics

**Additional Editor Comments (if provided):**

The manuscript has been reviewed by four reviewers. The reviewers were generally very enthusiastic and interested in the topic of this study. However they also had some concerns about the current manuscript especially relating to the technical analysis of the paper (noted by reviewers 2,3,4). There were also several comments where clarity could be greatly improved. In the end we think addressing these issues warrants a major revision. Based on the reviews, we will not be able to accept this version of the manuscript, but we would be willing to review a much-revised version that fully addresses each of the points raised by the reviewers.

**Journal Requirements:**

If the reviewer comments include a recommendation to cite specific previously published works, please review and evaluate these publications to determine whether they are relevant and should be cited. There is no requirement to cite these works unless the editor has indicated otherwise.

1) Please ensure that the CRediT author contributions listed for every co-author are completed accurately and in full.

At this stage, the following Authors/Authors require contributions: Yann Dussert, James S Borrell, Jonathan Stocks, Harriet V Hunt, Oliver W White, Paul Wilkin, Richard Buggs, Lucie Büchi, Sebsebe Demissew, Feleke Woldeyes, Ilia J Leitch, Wendawek M Abebe, and Richard A Nichols. Please ensure that the full contributions of each author are acknowledged in the "Add/Edit/Remove Authors" section of our submission form.

The list of CRediT author contributions may be found here: https://journals.plos.org/plosgenetics/s/authorship#loc-author-contributions

2) We ask that a manuscript source file is provided at Revision. Please upload your manuscript file as a .doc, .docx, .rtf or .tex. If you are providing a .tex file, please upload it under the item type u2018LaTeX Source Fileu2019 and leave your .pdf version as the item type u2018Manuscriptu2019.

3) Please upload all main figures as separate Figure files in .tif or .eps format. For more information about how to convert and format your figure files please see our guidelines:

https://journals.plos.org/plosgenetics/s/figures

4) We have noticed that you have uploaded Supporting Information files, but you have not included a list of legends. Please add a full list of legends for your Supporting Information files after the references list.

5) In the online submission form, you indicated that your data will be submitted to a repository upon acceptance. We strongly recommend all authors deposit their data before acceptance, as the process can be lengthy and hold up publication timelines. Please note that, though access restrictions are acceptable now, your entire minimal dataset will need to be made freely accessible if your manuscript is accepted for publication. This policy applies to all data except where public deposition would breach compliance with the protocol approved by your research ethics board. If you are unable to adhere to our open data policy, please kindly revise your statement to explain your reasoning and we will seek the editor's input on an exemption.

6) Some material included in your submission may be copyrighted. According to PLOS's copyright policy, authors who use figures or other material (e.g., graphics, clipart, maps) from another author or copyright holder must demonstrate or obtain permission to publish this material under the Creative Commons Attribution 4.0 International (CC BY 4.0) License used by PLOS journals. Please closely review the details of PLOS's copyright requirements here: PLOS Licenses and Copyright. If you need to request permissions from a copyright holder, you may use the PLOS Content Copyright Permission Form Please respond directly to this email and provide any known details concerning your material's license terms and permissions required for reuse, even if you have not yet obtained copyright permissions or are unsure of your material's copyright compatibility. Once you have responded and addressed all other outstanding technical requirements, you may resubmit your manuscript within Editorial Manager. Potential Copyright Issues:

i) Figures 1C, and S3. Please (a) provide a direct link to the base layer of the map (i.e., the country or region border shape) and ensure this is also included in the figure legend; and (b) provide a link to the terms of use / license information for the base layer image or shapefile. We cannot publish proprietary or copyrighted maps (e.g. Google Maps, Mapquest) and the terms of use for your map base layer must be compatible with our CC BY 4.0 license. Note: if you created the map in a software program like R or ArcGIS, please locate and indicate the source of the basemap shapefile onto which data has been plotted. If your map was obtained from a copyrighted source please amend the figure so that the base map used is from an openly available source. Alternatively, please provide explicit written permission from the copyright holder granting you the right to publish the material under our CC BY 4.0 license. If you are unsure whether you can use a map or not, please do reach out and we will be able to help you. The following websites are good examples of where you can source open access or public domain maps: * U.S. Geological Survey (USGS) - All maps are in the public domain. (http://www.usgs.gov) * PlaniGlobe - All maps are published under a Creative Commons license so please cite u201cPlaniGlobe, http://www.planiglobe.com, CC BY 2.0u201d in the image credit after the caption. (http://www.planiglobe.com/?lang=enl) * Natural Earth - All maps are public domain. (http://www.naturalearthdata.com/about/terms-of-use/).

**Reviewers' comments:**

Reviewer's Responses to Questions

**Comments to the Authors:**

**Please note here if the review is uploaded as an attachment.**

Reviewer #1: Review of ‘recurrent evolution of cryptic triploids in cultivated enset increases yield’

In this paper, the authors state that, prior to their current study, enset was thought to be exclusively diploid. Enset (Ensete ventricosum, related to banana), the pseudostem and leaves of which are used as a main food source in Ethiopia, is an under-utilized and under researched orphan crop which nevertheless has great potential to enhance food security in sub-Saharan Africa. Here, the authors, using a newly assembled chromosome-scale reference genome, and sequence data from 723 wild and domesticated enset individuals from southwestern Ethiopia, show that, unexpectedly, around 20% of cultivated enset clones are triploid. The authors tested whether enset triploids offered yield advantages to farmers and concluded that indeed they do and that triploid cytotypes have been systematically (but unknowingly) selected by farmers because of trait differences (thicker pseudostems thus higher yield in triploids).

The study also showed that the triploids originated from multiple independent triploidization events, likely originating from sexual crosses between diploid domesticated individuals. Most of the triploid lineages were not related to each other and had different diploid parents.

All wild individuals that were studied and sequenced on the contrary, were diploid. Among cultivated individuals, 414 were diploid and 214 were triploid.

I truly enjoyed reading this paper. I think this paper is particularly interesting because:

- The results present a correlation between clonality and polyploidy, as has been observed in other angiosperms: this clonal agricultural propagation allowed avoiding the costs of infertility, resulting from polyploidy/triploidy. In this case, the advantages of being polyploid outweigh the disadvantages/cost of polyploids (more competition in the wild, then in an agricultural setting). This would also explain why all enset individuals sampled in the wild, were diploids.

- This study is a very nice and prime example of the ‘trajectory of polyploidization’ in relation to domestication in real time, and explaining/showcasing how, throughout time (going back 10.000 years) other crops as well probably have been domesticated (through the selection of polyploids because of trait differences, i.e., bigger fruits, bigger ears, bigger seeds, …).

- It tries to explain – a little speculative, but making sense, in my opinion – the latitudinal gradient for the presence of triploids.

- The paper also explains why selecting for triploids (because of higher yield, for instance) might not necessarily be a good thing on the long-term, because of decreased genetic diversity (due to a small number of triploid lineages being dominant).

I have gone over the paper twice but could not come up with any major concerns. In my opinion, this is an interesting study on a very interesting, and unexpected finding. Unexpected, but when considering what we know about polyploidy and the many polyploid crops that have been selected for since the domestication of food crops, also maybe not completely unexpected.

Reviewer #2: This manuscript presents a compelling study on the recurrent evolution of triploidy in cultivated enset, demonstrating its association with increased yield and providing insights into polyploidy during domestication. The use of a new genome assembly and

large-scale resequencing is a strength, revealing unexpected triploidy in a species previously thought diploid. The integration of genomic, evolutionary, and phenotypic data is innovative, with implications for breeding and food security in Africa. The dataset is rich and the analyses are generally well executed, but several aspects of the study require clarification or additional support.

Line 119-128: The sequenced individual shows high genome-wide heterozygosity

(1.10%), yet a pseudo-haploid assembly was produced. Why was a phased haplotype-resolved assembly not attempted, especially given the potential for allelic differences in triploids? This could improve variant calling accuracy in heterozygous regions.

Line 138: Ploidy determination is crucial for this study, as it underpins the identification of triploid lineages and their implications for yield and domestication. Currently, it relies on conventional genomic survey methods: allele balance (AB) and k-mer spectra analyzed via Smudgeplot, with confirmation in six resequenced individuals. If possible, providing more accurate flow cytometry results using fresh samples for representative individuals would strengthen the validation.

Lines 163-167: Triploids are more common in southern sites, correlated with latitude but not altitude. Is this gradient potentially influenced by unmeasured factors like disease prevalence or cultural preferences?

Lines 170–178; 501–507: Several key conclusions of the manuscript rely on the

inferred number of clonal lineages, the reduced diversity of triploids, and the

predominance of a single triploid clone. However, because the clone-delimitation threshold was chosen visually and applied separately to diploids and triploids, these inferences may be sensitive to threshold choice. The authors should assess whether the main conclusions are robust across a reasonable range of clone-delimitation thresholds.

Lines 195-222: The low relatedness between diploid and triploid lineages suggests missing progenitors. Is this potentially due to incomplete sampling across Ethiopia, or could ancient triploidization events explain it? Additional phylogenetic modeling (e.g., using coalescent simulations) might clarify the timing of these events.

Lines 230-245: The Bayesian mixed-effects model shows triploids have 42–75% larger pseudostem volumes, but measurements were conducted in situ. It remains unclear how confounders such as soil fertility or farmer-specific practices were fully

accounted for beyond bioclimatic PCA. Given the impracticality of common garden

experiments for enset due to its long growth cycle, the authors might consider

assembling panels of geographically proximate diploid and triploid individuals

sharing similar environmental conditions to repeat the yield comparison, which

would help isolate ploidy effects from environmental heterogeneity. And the evidence supporting a yield increase associated with triploidy is not yet conclusive. I therefore suggest that the authors moderate this claim and remove “increase yield” from the title.

Minor points:

- Line 25: I think “polyploids” is sufficiently familiar to the audience and does not require explanation in parentheses.

- Line 81: the citations of the seven referred studies is missing.

- Line 214: estimation of kinship among individuals of different levels of ploidy is not common task, the author should briefly explain methods used and add citations. And citations should also be added in figure legend of 2B.

Reviewer #3: The manuscript describes the development of an improved long read assembly for an orphan crop, enset, a clonally propagated starch crop, which sustains a significant population in Ethiopia. Notably the authors have determined that what was believed to be a purely diploid species has domesticated triploid lineages among its ranks. This is a novel and interesting finding; the non-random distribution of the triploid lines across the different latitudes is clear and provides a nice discussion point. The authors are left to speculate as to when these triploid clones appeared, would it be possible to carry out Ks analyses of their limited whole genome resequencing data to attempt to gain some insights into this?

I have listed a few comments and questions related to the results and materials and methods below.

Results

The results indicates that the genome was sequenced from the seed of a cultivated variety Maze, yet in the M&M it is stated that the line sequenced was derived from a cross between Maze and an unknown line, does that mean the genome is for an F1 line? The assembly based on the limited information provided in the supplementary data seems excellent considering the relatively low PacBio coverage and the apparent heterozygosity in the sequenced line.

The data analyses appears robust but I did wonder about the genomic distribution of SNP loci after filtering? Did they see any biased selection for particular regions of the genome in the different lineages?

The statement ‘Thus farmers unconsciously classify plants according to their ploidy based on phenotype alone’ is a little misleading. It appears the farmers classify all their plants based on phenotype, so they will separate all plants, irrespective of ploidy, based on their phenotype.

Line 190: Maze in T2 is identified as a triploid clone. I assume this is different from the Maze line that was sequenced, which was a diploid?

The authors discuss the possible link between the increased ploidy and increased yield; although the phenotype data is not perfect for all the reasons they listed, I did wonder if the triploid clone which appeared to be predominantly cultivated (36% of the triploids) confirmed this theory – was the pseudostem much larger in these lines?

Materials and Methods

The methodology for the generation of the Omni-C library is generic and does not provide sufficient detail, perhaps provide a reference or indicate that this was completed by a service provider.

What was the length of the Illumina reads for the Omni-C data, presumably they were paired-end?

What was the age of the plants used to generate the RNASeq data? They were grown in a greenhouse?

How much RNASeq data was generated?

HiRise – version?

How were the Omni-C reads pre-processed?

Omni-C reads aligned with bwa – with default parameters, version?

Reviewer #4: This manuscript presents a comprehensive population genomic analysis of Ensete ventricosum, supported by a newly-assembled chromosome-scale reference genome and extensive sampling (723 wild and domesticated enset individuals). The authors report the unexpected presence of widespread cryptic triploid lineages in cultivated enset and demonstrate that triploidy is associated with increased yield. The study is novel and highly relevant for understanding crop domestication and food security in Sub-Saharan Africa. However, several aspects require clarification and improvement, particularly regarding the interpretation of triploid origins, ecological explanations, and the logical organization of the results and discussion sections. To further enhance your manuscript, we offer the following suggestions:

Major:

1.Mechanisms of triploid origin: The authors conclude that triploids arose from “multiple independent events among cultivated diploids” (lines 220-221). While the population genetic analyses support multiple origins, the underlying mechanism remains unclear. It is essential to further discuss whether these triploids originated via unreduced (2n) gametes within diploid populations or through hybridization between individuals of different ploidy levels. At present, the interpretation remains largely inferential; a more cautious discussion or additional allele dosage analysis would significantly strengthen this conclusion.

2.Limited explanation for the latitudinal gradient in triploid frequency: The study identifies a strong correlation between triploid frequency and latitude (lines 163-166), but the explanations provided (e.g., temperature effects on unreduced gamete formation, differences in farming practices) remain speculative and are not directly supported by data. The authors are encouraged to either provide additional analyses linking specific environmental variables to triploid frequency or clearly frame these explanations as hypotheses rather than definitive conclusions.

3.Genotype-environment interaction: The manuscript shows that triploids may have higher pseudostem volume (line 230), but it remains unclear whether this advantage is consistent across environments. It would be valuable to discuss whether triploids exhibit environment-specific advantages, their phenotypic stability across different altitudes, and the potential for genotype × environment interactions.

4.Genetic diversity and agricultural risk: The authors note that the dominance of a few triploid clones may reduce genetic diversity (lines 319-324). This is an important point but could be expanded. The discussion would benefit from a more explicit comparison with other clonally propagated crops.

5.Structure and logical flow of results and discussion: The results section would benefit from improved logical organization, with clearer connections between figures and findings. Similarly, the discussion is somewhat fragmented, with limited progression between paragraphs. The current structure relies heavily on simple transitions (e.g., “First”, “Finally”) rather than developing a cohesive narrative. The authors are strongly encouraged to strengthen the logical flow, enhance transitions between core ideas, and more effectively align the discussion with the study's primary findings to highlight their broader implications.

Minor points:

1.Language and grammar:

a)Line 26: “robustness to stress and to disease” → “robustness to stress and disease”

b)Line 38: “sub-saharan Africa” → “Sub-Saharan Africa”

c)Line 41: “a species entire genome” → “a species’ entire genome”

d)Line 65: “masking of deleterious mutations” → “masking by deleterious mutations”

e)Line 72: “polyploidy crops” → “polyploid crops”

f)Line 98: simplify to “a high-yielding, ecologically resilient and flexible crop”

g)Line 145: “suggests” → “suggested”; “not selfing” → “rather than selfing”

h)Line 257: “are possible confounding effects” → “were possible confounding effects”

i)Line 259: “differences of” → “differences in”

2.Formatting and notation:

a)Line 101: “1,500 to 3,000 m” → “1,500-3,000 m”

b)Ensure consistent formatting of k-mer throughout the manuscript

c)Italicize all p-values (e.g., lines 164, 166, 167, 187)

d)Define abbreviations such as FST and CI at first mention

e)Line 357: “DNAse I” → “DNase I”

f)Ensure consistent use of quotation marks (e.g., lines 99 and 555)

3.Terminology consistency:

a)Use “clonal propagation” consistently instead of alternating with “vegetative propagation”

b)Ensure consistent usage of key technical terms throughout the manuscript

c)Standardize formatting conventions (e.g., gene names in italics)

4.Figures and presentation:

a)Provide clearer explanation of interaction effects in Figure 3

b)Strengthen connections between figures and corresponding text in the results section

5.References:

Please ensure that all references strictly follow the journal’s formatting guidelines.

**********

**Have all data underlying the figures and results presented in the manuscript been provided?**

Large-scale datasets should be made available via a public repository as described in the *PLOS Genetics*  data availability policy, and numerical data that underlies graphs or summary statistics should be provided in spreadsheet form as supporting information.

Reviewer #1: Yes

Reviewer #2: Yes

Reviewer #3: Yes

Reviewer #4: None

**********

PLOS authors have the option to publish the peer review history of their article (what does this mean?). If published, this will include your full peer review and any attached files.

If you choose “no”, your identity will remain anonymous but your review may still be made public.

**Do you want your identity to be public for this peer review?**  For information about this choice, including consent withdrawal, please see our Privacy Policy.

Reviewer #1: No

Reviewer #2: No

Reviewer #3: No

Reviewer #4: No

**Figure resubmission:**

While revising your submission, we strongly recommend that you use PLOS’s NAAS tool (https://ngplosjournals.pagemajik.ai/artanalysis) to test your figure files. NAAS can convert your figure files to the TIFF file type and meet basic requirements (such as print size, resolution), or provide you with a report on issues that do not meet our requirements and that NAAS cannot fix.

After uploading your figures to PLOS’s NAAS tool - https://ngplosjournals.pagemajik.ai/artanalysis, NAAS will process the files provided and display the results in the "Uploaded Files" section of the page as the processing is complete. If the uploaded figures meet our requirements (or NAAS is able to fix the files to meet our requirements), the figure will be marked as "fixed" above. If NAAS is unable to fix the files, a red "failed" label will appear above. When NAAS has confirmed that the figure files meet our requirements, please download the file via the download option, and include these NAAS processed figure files when submitting your revised manuscript.

**Reproducibility:**

To enhance the reproducibility of your results, we recommend that authors of applicable studies deposit laboratory protocols in protocols.io, where a protocol can be assigned its own identifier (DOI) such that it can be cited independently in the future. Additionally, PLOS ONE offers an option to publish peer-reviewed clinical study protocols. Read more information on sharing protocols at https://plos.org/protocols?utm_medium=editorial-email&utm_source=authorletters&utm_campaign=protocols

# Author comment: Author response to Decision Letter 1

**DOI:** 10.1371/journal.pgen.1012241.r002

18 Jun 2026

Attachment Submitted filename: TriploidEnsetMS_PlosGenetics_responseReviews.docx

# Peer review: Decision Letter 1

**DOI:** 10.1371/journal.pgen.1012241.r003

**Authors:** Justin Fay, Jae Young Choi

6 Jul 2026

Dear Dr Dussert,

We are pleased to inform you that your manuscript entitled "Recurrent evolution of cryptic triploids in cultivated enset increases yield" has been editorially accepted for publication in PLOS Genetics. Congratulations!

Before your submission can be formally accepted and sent to production you will need to complete our formatting changes, which you will receive in a follow up email. Please be aware that it may take several days for you to receive this email; during this time no action is required by you. Please note: the accept date on your published article will reflect the date of this provisional acceptance, but your manuscript will not be scheduled for publication until the required changes have been made.

Once your paper is formally accepted, an uncorrected proof of your manuscript will be published online ahead of the final version, unless you’ve already opted out via the online submission form. If, for any reason, you do not want an earlier version of your manuscript published online or are unsure if you have already indicated as such, please let the journal staff know immediately at plosgenetics@plos.org.

In the meantime, please log into Editorial Manager at https://www.editorialmanager.com/pgenetics/, click the "Update My Information" link at the top of the page, and update your user information to ensure an efficient production and billing process. Note that PLOS requires an ORCID iD for all corresponding authors. Therefore, please ensure that you have an ORCID iD and that it is validated in Editorial Manager. To do this, go to ‘Update my Information’ (in the upper left-hand corner of the main menu), and click on the Fetch/Validate link next to the ORCID field.  This will take you to the ORCID site and allow you to create a new iD or authenticate a pre-existing iD in Editorial Manager.

If you have a press-related query, or would like to know about making your underlying data available (as you will be aware, this is required for publication), please see the end of this email. If your institution or institutions have a press office, please notify them about your upcoming article at this point, to enable them to help maximise its impact. Inform journal staff as soon as possible if you are preparing a press release for your article and need a publication date.

Thank you again for supporting open-access publishing; we are looking forward to publishing your work in PLOS Genetics!

Yours sincerely,

Jae Young Choi

Academic Editor

PLOS Genetics

Justin Fay

Section Editor

PLOS Genetics

Aimée Dudley

Editor-in-Chief

PLOS Genetics

Anne Goriely

Editor-in-Chief

PLOS Genetics

www.plosgenetics.org

BlueSky: @plos.bsky.social

----------------------------------------------------

Comments from the reviewers (if applicable):

Reviewer's Responses to Questions

**Comments to the Authors:**

**Please note here if the review is uploaded as an attachment.**

Reviewer #2: The authors have addressed my questions, and there is no more question.

Reviewer #4: The authors has addressed all my concerns. I would like to congratulate the authors for this timely contribution.

**********

**Have all data underlying the figures and results presented in the manuscript been provided?**

Large-scale datasets should be made available via a public repository as described in the *PLOS Genetics*  data availability policy, and numerical data that underlies graphs or summary statistics should be provided in spreadsheet form as supporting information.

Reviewer #2: Yes

Reviewer #4: None

**********

PLOS authors have the option to publish the peer review history of their article (what does this mean?). If published, this will include your full peer review and any attached files.

If you choose “no”, your identity will remain anonymous but your review may still be made public.

**Do you want your identity to be public for this peer review?**  For information about this choice, including consent withdrawal, please see our Privacy Policy.

Reviewer #2: No

Reviewer #4: No

----------------------------------------------------

**Data Deposition**

If you have submitted a Research Article or Front Matter that has associated data that are not suitable for deposition in a subject-specific public repository (such as GenBank or ArrayExpress), one way to make that data available is to deposit it in the Dryad Digital Repository. As you may recall, we ask all authors to agree to make data available; this is one way to achieve that. A full list of recommended repositories can be found on our website.

The following link will take you to the Dryad record for your article, so you won't have to re‐enter its bibliographic information, and can upload your files directly:

http://datadryad.org/submit?journalID=pgenetics&manu=PGENETICS-D-26-00202R1

More information about depositing data in Dryad is available at http://www.datadryad.org/depositing. If you experience any difficulties in submitting your data, please contact help@datadryad.org for support.

Additionally, please be aware that our data availability policy requires that all numerical data underlying display items are included with the submission, and you will need to provide this before we can formally accept your manuscript, if not already present.

----------------------------------------------------

**Press Queries**

If you or your institution will be preparing press materials for this manuscript, or if you need to know your paper's publication date for media purposes, please inform the journal staff as soon as possible so that your submission can be scheduled accordingly. Your manuscript will remain under a strict press embargo until the publication date and time. This means an early version of your manuscript will not be published ahead of your final version. PLOS Genetics may also choose to issue a press release for your article. If there's anything the journal should know or you'd like more information, please get in touch via plosgenetics@plos.org.

# Editor report: Acceptance letter

**DOI:** 10.1371/journal.pgen.1012241.r004

**Authors:** Justin Fay, Jae Young Choi

PGENETICS-D-26-00202R1

Recurrent evolution of cryptic triploids in cultivated enset increases yield

Dear Dr Dussert,

We are pleased to inform you that your manuscript entitled "Recurrent evolution of cryptic triploids in cultivated enset increases yield" has been formally accepted for publication in PLOS Genetics! Your manuscript is now with our production department and you will be notified of the publication date in due course.

The corresponding author will soon be receiving a typeset proof for review, to ensure errors have not been introduced during production. Please review the PDF proof of your manuscript carefully, as this is the last chance to correct any errors. Please note that major changes, or those which affect the scientific understanding of the work, will likely cause delays to the publication date of your manuscript.

Soon after your final files are uploaded, unless you have opted out or your manuscript is a front-matter piece, the early version of your manuscript will be published online. The date of the early version will be your article's publication date. The final article will be published to the same URL, and all versions of the paper will be accessible to readers.

For Research Articles, you will receive an invoice from PLOS for your publication fee after your manuscript has reached the completed accept phase. If you receive an email requesting payment before acceptance or for any other service, this may be a phishing scheme. Learn how to identify phishing emails and protect your accounts at https://explore.plos.org/phishing.

Thank you again for supporting PLOS Genetics and open-access publishing. We are looking forward to publishing your work!

With kind regards,

Kannan R K Kuppusamy, B.TECH BIOTECHNOLOGY

PLOS Genetics

On behalf of:

The PLOS Genetics Team

Carlyle House, Carlyle Road, Cambridge CB4 3DN | United Kingdom

plosgenetics@plos.org | +44 (0) 1223-442823

plosgenetics.org | Twitter: @PLOSGenetics
